# RAS Inhibitors: Changing the Paradigm from the Undruggable

**DOI:** 10.3390/pharmaceutics18070773

**Published:** 2026-06-24

**Authors:** Federica Campana, Emanuela De Bellis, Floriana Porcaro, Gaetano Di Guida, Luigi Della Gravara, Alberto Servetto, Floriana Morgillo, Federica Papaccio, Amelia Filippelli, Carminia Maria Della Corte, Valeria Conti

**Affiliations:** 1 Department of Human, Philosophical and Formation Sciences, University of Salerno, 84084 Fisciano, Italy; 2 PhD School “Clinical and Translational Oncology (CTO)”, Scuola Superiore Meridionale, University of Naples “Federico II”, 80131 Naples, Italy; 3 Department of Clinical Medicine and Surgery, University of Naples “Federico II”, 80131 Naples, Italy; 4 Department of Precision Medicine, University of Campania “Luigi Vanvitelli”, 80131 Naples, Italy; 5 Department of Medicine, Surgery, and Dentistry, Scuola Medica Salernitana, University of Salerno, 84081 Baronissi, Italy; 6 Clinical Pharmacology Unit, San Giovanni di Dio e Ruggi d’Aragona University Hospital, 84100 Salerno, Italy

**Keywords:** RAS-inhibitors, RAS mutations, solid tumors

## Abstract

For decades RAS mutations represented a chimera for drug developers, as all efforts put into attempting to pharmacologically inhibit them failed. Finally, in recent years, the advent of the KRAS G12C inhibitors sotorasib and adagrasib pushed the development of several new drugs; therefore, the landscape of RAS inhibitors evolved and now includes several compounds with different mechanisms of action. In this review we provide an updated summary of RAS inhibitor drugs, detailing their mechanism of action, pharmacokinetics and toxicity profile as well as efficacy studies.

## 1. Introduction

Despite being highly prevalent in several solid tumors, RAS oncogenic mutations represented for many decades the prototype of the undruggable gene alteration. Recently, the development of KRAS G12C inhibitors opened the way to KRAS inhibitors.

This review aims at summarizing the current field of RAS inhibitors.

### RAS Alterations in Cancer

Rat sarcoma (RAS) genes encode four highly homologous, yet functionally non-redundant, 21 kDa membrane-bound small monomeric GTPases, which act as molecular switches that transmit signals from membrane receptors to various downstream effector molecules playing an important role in cell survival, metabolism, adhesion, migration, differentiation, cell-cycle entry, cytoskeletal dynamics and cancer proliferation, since they are the most frequently mutated oncogenes in various types of human cancer [1].

RAS proteins are composed of two main domains: a highly conserved catalytic domain, namely the G domain, and a hypervariable region (HVR). The catalytic G domain consists of three regions: the GTP-binding region, as well as the switch-I and switch-II regions, and is responsible for engaging with their main downstream effectors. The HVR comprises the CAAX motif, which has a key role in plasma localization. Currently, differences in RAS isoforms have been ascribed largely to sequence differences within their C-terminal HVRs, a site at which RAS proteins are differentially lipid-modified, suggesting distinct mechanisms of plasma membrane localization and subcellular trafficking [2].

The catalytic G domain is responsible for nucleotide exchange, and RAS proteins normally cycle between an inactive GDP-bound OFF-state, a transient nucleotide-free state, and an active GTP-bound ON-state, which engages effectors to initiate various signaling cascades. The balance between these states is regulated by guanine nucleotide-exchange factors (GEFs), such as SOS, which catalyze the exchange of GDP for GTP, and GTPase-activating proteins (GAPs), such as neurofibromin 1 (NF1), which enhance RAS’s relatively poor intrinsic GTPase activity to speed up GTP hydrolysis [1].

Once activated, RAS proteins undergo a conformational change in the switch-I and switch-II domains, enabling them to interact with multiple downstream intracellular pathways. One of the most well-defined pathways is the fibrosarcoma (RAF)-mitogen-activated protein kinase (MEK)-extracellular-regulated protein kinase (ERK) signaling cascade, which plays a critical role in regulating cell proliferation, differentiation, migration, and survival. KRAS also plays a key role in the PI3K-AKT-mammalian target of the rapamycin (mTOR) signaling pathway, which is involved in the regulation of cell proliferation, differentiation, apoptosis, glucose transport and other cellular life activities. Ral-GDS is another key effector of KRAS, which promotes the transition of RAL from a GDP-bound to a GTP-bound state, and downstream signaling, which promotes cell-cycle progression. Downstream effectors of RAL proteins include phospholipase D (PLD) associated with endocytosis, TANK-binding kinase 1 (TBK1) associated with viral immunity, and cell division cycle 42 (CDC42) associated with cell migration. The Ral-GDS-RALPLD pathway plays an important role in KRAS-driven tumorigenesis, especially in the regulation of vesicle transport and cytoskeletal organization [3].

KRAS is also linked to additional signaling pathways. It has been shown that recombinant T-cell lymphoma invasion and metastasis inducing protein 1 (TIAM1) is a downstream effector of KRAS proteins, which acts as a RAS-related C3 botulinum toxin substrate 1 (RAC1)-specific GEF to promote RAC binding to GTP to activate the RAC1 signaling pathway that affects cell migration, adhesion, actin protein cytoskeleton formation, endocytosis, and membrane transport. In addition, KRAS proteins can also regulate the phospho-acylinositol signaling pathway through activation of phosphatidylinositol-specific phospholipase C ε (PLCε). PLCε is a key signaling enzyme that hydrolyzes PIP2 to generate inositol trisphosphate (IP3), which stimulates an increase in intracellular Ca^2+^ and activation of proteins kinase C (PKC). Moreover, potential downstream effectors of RAS may include the novel RAS effector 1A (NORE1A), Af6, RAS and Rab interactor 1 (RIN1), and growth factor receptor 14 (Grb14) [3].

In conclusion, the signaling pathways regulated by KRAS are intricate and multifaceted, influencing various cell processes such as cell proliferation, differentiation, migration, and apoptosis. The diverse downstream effects of KRAS mutations contribute significantly to tumorigenesis, making KRAS an important target for therapeutic intervention in cancers such as lung adenocarcinoma [3].

**Figure 1:**
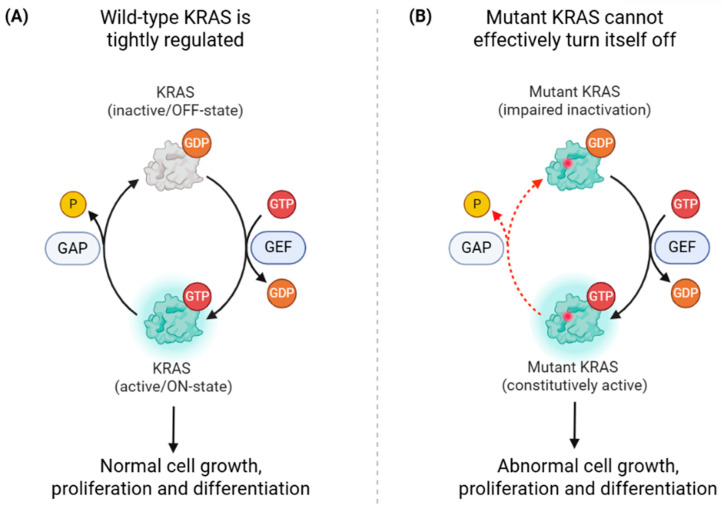
Structure and function of a KRAS protein. (**A**) Under physiological conditions, the KRAS protein cycles between the active KRAS-GTP ON-state and the inactive KRAS-GDP OFF-state, regulated by the exchange and hydrolysis of GTP/GDP, controlling critical cellular processes such as cell proliferation, apoptosis, differentiation, survival, and migration. The KRAS inactive protein releases GDP upon interaction with guanine nucleotide-exchange factors (GEFs) and rapidly binds to GTP, transitioning into the active KRAS-GTP ON-state. The KRAS protein itself has GTPase activity, which is augmented by GTPase-activating proteins (GAPs), thereby catalyzing the conversion of KRAS-GTP back to the inactive KRAS-GDP OFF-state. (**B**) The presence of KRAS point mutations compromises the intrinsic GTPase of KRAS and GAP activities, leading to the progressive accumulation of the active KRAS-GTP protein, constitutive activation of downstream signaling pathways, and subsequent promotion of tumor growth. Created in BioRender. Filippelli, A. (2026), https://biorender.com/r2gmi0h.

In the presence of oncogenic RAS mutations or gene amplifications, however, the closely regulated cycling of RAS is disrupted, leading to uncontrolled activation of downstream signaling and cell growth (see Figure 1) [1].

More than 90% of RAS oncogenic mutations are characterized by single-base missense mutations and occur at several ‘hotspots’, such as at codon 12 in exon 2 (G12) and codon 13 (G13) and 61 (Q61). These mutations cause a conformational change in the catalytic G domain of RAS proteins, impairing their GTPase activity and interaction with GAPs. This shifts the RAS equilibrium towards the active GTP-bound ON-state, consecutively activating different downstream effector pathways [1,4].

There are three major RAS isoforms: Kirsten rat sarcoma (KRAS), Harvey rat sarcoma (HRAS) and neuroblastoma rat sarcoma (NRAS). KRAS has two splicing isoforms: a minor 4A (KRAS-4A) and a major 4B (KRAS-4B). Among RAS genes, KRAS is the most frequently mutated in cancer (85%), followed by NRAS (12%) and HRAS (3%) [1].

KRAS mutations are known drivers of the most lethal cancers and are implicated in 25–30% of all human cancers, in about 19% of non-small-cell lung cancers (NSCLCs), about 40% of Colorectal Cancers (CRCs), and about 73% of pancreatic ductal adenocarcinoma (PDAC) cases, with the glycine-to-cysteine substitution at codon 12 (G12C) representing a druggable target in human solid tumors. KRAS G12C mutations are less common in solid tumors other than NSCLC and CRC, where their incidence is approximately 14% and 3.6%, respectively, in Western countries [5,6].

Cigarette smoking is strongly associated with the development of KRAS mutations. In general, the genetic pattern differs substantially between smokers and non-smokers: KRAS G12C is typically associated with smokers, whereas KRAS G12D is more commonly found in non-smokers [2].

These oncogenic mutations are activating mutations and lead to constitutive activation of the GTP-bound form of the KRAS protein, resulting in increased stimulation of cellular proliferation. Activation of the downstream signal transduction pathways occurs through cross-talk with other growth factor receptors, such as members of the EGFR receptor family, mediated by adaptor proteins including SOS1. Additionally, co-mutations such as STK11, KEAP1, and TP53 are frequently detected, making the KRAS mutational landscape highly heterogeneous [2,7].

In 2013, the groundbreaking discovery of a drug-binding pocket on the surface of mutant RAS, known as the switch-II pocket, which was only accessible in the GDP-bound state, enabled the development of the first preclinical compounds that selectively and with high affinity bind to the chemically reactive cysteine residue of the KRAS G12C mutant, while sparing wild-type RAS proteins, which lack reactive cysteine residues. By forming a covalent bond, these agents lock the mutant KRAS G12C protein in its GDP-bound OFF-state, thereby effectively preventing downstream signaling activation [4] and thereby opening the field of KRAS inhibitors.

## 2. Current Landscape of KRAS Inhibitors: Pharmacology and Toxicity Profile

### 2.1. Targeting KRAS in Cancer

The difficulty in developing RAS inhibitors arises from two main factors. First, RAS exhibits an extremely high affinity for its ligand GTP (in the picomolar range), making it poorly competitive with alternative ligands. Second, no well-defined binding pocket had been identified that could accommodate inhibitory molecules capable of altering its conformation. As a result, alternative strategies were required.

Pharmaceutical chemists therefore focused on the KRAS G12C variant, an isoform generated by the substitution of glycine with cysteine at codon 12. KRAS G12C inhibitors bind to a specific region of the protein known as the switch-II pocket, where they form covalent and irreversible bonds—often through disulfide bridge formation—thereby stabilizing the protein in its inactive, GDP-bound state [8].

#### 2.1.1. KRAS G12C Selective OFF-State Inhibitors

Sotorasib (AMG-510) is a first-in-class KRAS G12C OFF-state inhibitor which binds covalently and irreversibly to the cysteine residue of the KRAS G12C-mutant cell lines, without affecting wild-type KRAS: as a consequence, the KRAS protein is locked in an inactive state and its downstream signaling effects are blocked [9,10].

Preclinical evidence showed that sotorasib selectively impaired the viability of KRAS G12C-mutant lines, and coadministration with inhibitors of other signaling pathways afforded evidence for synergistic effects on cell viability [9,11]. In particular, preclinical studies investigating sotorasib involved KRAS G12C-mutant tumor cell lines (in vitro) as well as xenograft models (in vivo) [2,7].

Two independent research groups, using distinct methodological approaches, demonstrated the chemical–biological rationale underlying the use of sotorasib, namely the formation of an irreversible covalent bond mediated by the mutant cysteine residue. Specifically, the first group employed a strategy combining a cell-permeable prodrug with a chloroacetamide-modified GDP analog, whereas the second group utilized sulfonamide and acrylamide derivatives that bound allosterically to the switch-II pocket of KRAS—a region previously identified during efforts to develop non-covalent RAS inhibitors. Both groups concluded that covalent modification of the mutant cysteine residue resulted in stabilization of KRAS in its inactive GDP-bound state, thereby preventing downstream oncogenic signaling [12].

These early investigations led to the development of ARS-853, one of the first covalent inhibitors of KRAS G12C active in vitro. The transition from in vitro proof-of-concept to in vivo validation occurred with ARS-1620, which demonstrated improved potency, enhanced stability, and optimized pharmacokinetic (PK) properties [12].

On 28 May 2021, sotorasib received its first approval in the USA for the treatment of adult patients with KRAS G12C-mutant locally advanced or metastatic NSCLC. This indication came upon accelerated approval based on objective response rate (ORR) and duration of response (DOR), and its continued approval may be contingent upon verification and description of clinical benefit in confirmatory trials.

The drug is also indicated, in combination with panitumumab, for the treatment of adult patients with KRAS G12C-mutant metastatic CRC, as determined by an FDA-approved test, who have received prior fluoropyrimidine-, oxaliplatin- and irinotecan-based chemotherapy [9,10].

Adagrasib (MRTX849), the second mutant-selective covalent KRAS G12C OFF-state inhibitor to enter clinical development, is functionally similar to sotorasib with a substantially longer half-life [4].

Like sotorasib, adagrasib forms a covalent bond with cysteine 12 in the switch-II pocket, locking the mutant KRAS protein in an inactive state and inhibiting downstream signaling. In vitro studies demonstrate adagrasib’s potent and selective inhibition of KRAS-dependent signal transduction and cell viability [13]. With broad-spectrum antitumor activity observed across diverse cancer types, including lung, colon and pancreatic cancers, adagrasib induces significant tumor regression in preclinical models, showing minimal off-target effects [13].

Adagrasib potently inhibited tumor cell proliferation at nanomolar concentrations, indicating high affinity and favorable pharmacologic activity. Its antitumor effects were associated with suppression of ERK- and S6-dependent signaling, consistent with inhibition of the MAPK pathway. In addition, partial inhibition of the PI3K/AKT signaling cascade was observed. Maximal MAPK pathway suppression was achieved approximately 6 h after compound administration in murine models, at doses ≥ 30 mg/kg. Tumor regressions were dose-dependent and were complete in a substantial proportion of xenograft models [14].

Furthermore, adagrasib can reverse an immunosuppressive tumor microenvironment (TME), enhancing the presence of CD4+ and CD8+ T cells while reducing myeloid-derived suppressor cells [13].

On 12 December 2022, adagrasib received its first approval in the USA for the treatment of adults with KRAS G12C-mutated locally advanced or metastatic NSCLC, as determined by an FDA-approved test, who have received at least one prior systemic therapy: this indication was approved under accelerated approval-based ORR and DOR [15,16].

On 21 June 2024, the FDA granted accelerated approval to adagrasib plus cetuximab for adults with KRAS G12C-mutated locally advanced or metastatic CRC, as determined by an FDA-approved test, who have received prior treatment with fluoropyrimidine-, oxaliplatin-, and irinotecan-based chemotherapy.

Table 1 summarizes the pharmacological differences between adagrasib and sotorasib.

Multiple other next-generation covalent KRAS G12C OFF-state inhibitors are currently under early-phase clinical exploration in NSCLC and other malignancies, with the most mature data in Western patients existing for divarasib (GDC-6036), a highly selective inhibitor which irreversibly binds the KRAS protein by forming a covalent bond with cysteine 12 in the switch-II pocket [4], thereby preventing its transition to the active conformation.

Divarasib was over 18,000-fold more selective for mutant G12C than wild-type cell lines [17] and showed a dose-dependent target inhibition in multiple KRAS G12C-positive cell lines and xenograft mouse models, correlating to high antitumor potency. It also demonstrates greater potency and selectivity than sotorasib and adagrasib (5 to 20 times more potent and up to 50 times more selective in vitro) [13]. Specifically, in vitro, divarasib appears to be significantly more potent and selective than competing agents, to the extent that doses below 400 mg achieve inhibitory activity against cell proliferation in xenograft murine models and cancer cell lines [17].

Preclinical studies demonstrated a median half-maximal inhibitory concentration (IC50) in the sub-nanomolar range. The earliest clinical data published from Sacher et al. in a phase I study on the agent (NCT04449874) showed that, after a single 400 mg dose of divarasib, the average T1/2 was 17.6 h (±2.7 h). Steady state was assessed at cycle 1 day 8 or cycle 2 day 1. In total, 76 patients were evaluated at steady state and demonstrated mean maximum concentration was 657 nanograms per milliliter (±185) and mean area under the curve was 9130 nanograms x hours per milliliter (±3160) [17,18].

Its safety profile makes divarasib a potential agent that can be used both as a single agent and in combination with other anticancer therapies [13]. Adverse events were reported in 127 patients (93%) taking divarasib. This included 11% grade 3 and 1% grade 4 adverse events. There were no grade 5 events reported. The most common adverse events in the NSCLC cohort include nausea (*n* = 27, 78%), vomiting (*n* = 38, 63%), diarrhea (*n* = 36, 60%), and fatigue (*n* = 16, 27%). Dose reductions occurred in 19 (14%) and the agent was discontinued in 4 participants (3%) [17].

Fulzerasib (IBI351) is an oral small-molecule covalent inhibitor of KRAS G12C which shares the same mechanism of action as other agents in its class: it covalently and irreversibly modifies the cysteine residue of the KRAS G12C protein, inhibits GTP/GDP exchange, and locks the protein in an inactive state [19,20].

Fulzerasib demonstrated promising efficacy and durable response in patients with KRAS G12C-mutated advanced solid tumors (NSCLC comprising 94.3% of cases [166/176]) in a multicenter, open-label, phase Ia/Ib study conducted at 54 hospitals in China (NCT05005234). In this study fulzerasib, administered at a dose of 600 mg BID, was rapidly absorbed with a Tmax of approximately 2 h, and it was eliminated with a half-life of around 5 h after a single dose. Multiple-dose PK profiles were generally comparable with single-dose PK characteristics, and no accumulation was observed after multiple doses [21].

Treatment-emergent adverse events (TEAEs) occurred in 174 (98.9%) patients, while treatment-related adverse events (TRAEs) occurred in 168 (95.5%) patients. The most common TRAEs were anemia (47.7%), an increase in alanine aminotransferase (33%), and an increase in aspartate aminotransferase (31.3%). Grade 3 or higher TRAEs occurred in 64 (36.4%) patients, most frequently a gamma-glutamyl transferase increase (10.2%) and anemia (6.8%). TRAEs leading to dose interruption occurred in 63 patients (35.8%) mainly due to anemia (5.7%), asthenia (5.7%), alanine aminotransferase increase (4.5%), gamma-glutamyl transferase increase (4.0%), and aspartate aminotransferase increase (3.4%). TRAEs leading to dose reduction occurred in 33 patients (18.8%), mainly due to asthenia (3.4%), anemia (2.8%), and gamma-glutamyl transferase increase (2.3%). TRAEs leading to treatment discontinuation occurred in 5 patients (2.8%) due to gamma-glutamyl transferase increase, asthenia, pneumonia, and maculopapular rash. TEAEs leading to death occurred in 10 patients (5.7%), of which 2 (1.1%) deaths were suspected to be treatment-related as per investigator’s assessment [21].

Garsorasib (D-1553) acts as a potent, selective, and covalent inhibitor specifically targeting KRAS G12C. Similar to other inhibitors of KRAS G12C, it binds to the switch-II pocket, effectively locking the protein into its inactive, GDP-bound state. Preclinical studies have demonstrated that garsorasib strongly inhibits tumor growth, with high oral bioavailability and the ability to penetrate the central nervous system (CNS) [19].

A phase I multicenter clinical trial (NCT05383898) evaluated the safety, PK, and efficacy of garsorasib in patients with advanced KRAS G12C-mutated NSCLC through dose escalation and frequency adjustment [20]. Patients received garsorasib 600 mg orally once daily (QD), 800 mg QD, 1200 mg QD, 400 mg BID, or 600 mg BID with dose escalation. Across all dose levels, the Tmax was 1–4 h after a single oral dose and 1–2 h at steady state following repeated administration, with an elimination half-life of approximately 3–6 h. No drug accumulation was observed after 14 days of administration [19,22].

TRAEs occurred in 94.9% (75/79) of patients, with the most common being liver function abnormalities and gastrointestinal events, indicating that garsorasib has a favorable safety and tolerability profile. The incidence of grade ≥ 3 liver function abnormalities was 37.8% (14/37) in patients whose last anti-PD-L1 treatment was within 90 days before the initiation of garsorasib, compared with 16.7% (7/42) in those who had not received prior anti-PD-L1 treatment or whose last anti-PD-L1 treatment was at least 90 days before garsorasib initiation. These data suggest that this subset of patients has a higher potential risk of garsorasib-related hepatotoxicity, requiring more frequent monitoring of liver function [19].

Glecirasib (JAB-21822) is a novel covalent oral KRAS G12C inhibitor approved for marketing in China in 2025 for the treatment of NSCLC [6,19].

Compared with other molecules in the same class, glecirasib demonstrates substantially enhanced drug stability and oral bioavailability through novel modifications to the molecular scaffold, strategically regulating overall lipophilicity, constructing a new hydrogen bond network to enhance target-binding affinity, and incorporating multihalogen groups to block metabolically labile sites. Meanwhile, glecirasib redirects its primary elimination pathway to glucuronidation, distinguishing itself from agents of the same class: the structural modification not only reduces CYP-mediated toxicities and the risk of drug–drug interactions, but also identifies elevated bilirubin as a critical adverse event signal requiring close monitoring. These structural optimizations achieve an equilibrium between high potency, advantageous PK and minimal reliance on CYP enzymes, thereby providing support for its safe use in clinical settings.

Glecirasib demonstrated strong antitumor efficacy as a monotherapy, both in vitro and in vivo, in preclinical models. NCT05009329 was a first-in-human, open-label phase 1/2 study of glecirasib whose primary objective was to evaluate the safety and tolerability of the molecule in patients with advanced solid tumors harboring KRAS G12C mutations [23]. The trial includes a phase 1 dose escalation cohort, in which patients were enrolled in 5 different dose levels: 200 mg QD, 400 mg QD, 800 mg QD, 400 mg BID and 400 mg three times a day (TID), and a phase 2a dose expansion cohort. In phase 1, the most common TRAEs (≥10%) included anemia (24.5%), total bilirubin increase (20.8%), direct bilirubin increase (15.1%), proteinuria (13.2%) and indirect bilirubin increase (11.3%). In the QD cohorts, only grade 1 and 2 TRAEs were observed. Glecirasib was rapidly absorbed, with an average Tmax of 2 h and an elimination half-life of approximately 4.99–5.54 h [19,23].

#### 2.1.2. KRAS G12C Selective ON-State Inhibitors

KRAS G12C exhibits the highest intrinsic GTP hydrolysis rate among KRAS mutants; nevertheless, most of the protein remains in the active, GTP-bound ON-state. This supports the rationale for directly targeting KRAS G12C in its GTP-bound ON-state conformation [4].

Elironrasib (RMC-6291) is a first-in-class covalent tri-complex ON-state inhibitor that selectively targets the active GTP-bound state of KRAS G12C. In particular, elironrasib enters inside the cell and binds to the abundant intracellular chaperone protein cyclophilin A (CypA). This binary complex then binds to the RAS (ON) protein creating a new binding pocket on the protein surface, immediately adjacent to the G12 mutation hotspot. Elironrasib covalently modifies the cysteine at position 12, resulting in the formation of an irreversible inhibitory tri-complex in which CypA sterically occludes the RAS effector binding face, preventing downstream effectors from engaging RAS (ON) and thereby inhibiting its oncogenic signaling [24]. Through this mechanism, it may be possible to overcome resistance mechanisms that emerge following treatment with first-generation KRAS inhibitors [25]. Preliminary research data indicate that elironrasib can produce deep and persistent inhibition of the RAS pathway activity in cell lines previously treated with KRAS G12C (OFF) inhibitors that had developed resistance, demonstrating that KRAS G12C (ON) inhibitors may act as multiselective agents capable of targeting known resistance mechanisms [26].

In a phase 1 clinical trial, elironrasib has demonstrated encouraging preliminary clinical tolerability and efficacy in patients with KRAS G12C-mutant NSCLC and CRC, including an ORR of 50% in patients with NSCLC previously treated with KRAS G12C OFF-state inhibitors [4]. Patients received escalating doses of 50, 100, and 200 mg QD and 100, 200, 300, and 400 mg BID. Each cycle had 21 days, with efficacy assessed every 6 weeks. Elironrasib exhibited dose-dependent exposure with a median Tmax of 1.0 h and terminal half-life of 1.8 h. Modeling projected average target occupancy of ≥~90% at 100 mg BID and above [27].

The TRAEs occurring in ≥10% of patients were nausea (34%), diarrhea (30%), QTc prolongation (21%), vomiting and fatigue (15% each). The most common grade 3 TRAEs were QTc prolongation reported in five patients (3 at 400 mg BID, 1 at 300 mg, 1 at 200 mg BID). Only one out of these five patients had an average QTc interval > 501 ms. Most events of grade 3 QTc prolongation resolved to grade 1 or normal following dose interruption and/or reduction, and all five patients were asymptomatic and remained on treatment at a reduced dose. No treatment-related grade ≥ 3 hepatotoxicity was observed. No patients experienced a treatment-related grade 4 or 5 AE, or a treatment-emergent AE that led to treatment discontinuation [27].

Based on these data, elironrasib was recently granted breakthrough therapy designation by the FDA for the treatment of patients with advanced KRAS G12C-mutant NSCLC who have received standard first-line therapies [4].

#### 2.1.3. KRAS G12C Selective Dual (ON/OFF)-State Inhibitors

Preliminary data supports the hypothesis that targeting both the ON- and OFF- forms of KRAS G12C results in greater potency, deeper responses and slowed development of resistance, leading to significant benefits over approved, (OFF)-only KRAS G12C inhibitors [28].

BBO-8520 is a first-in-class covalent dual inhibitor which covalently binds to the switch-II pocket of KRAS G12C and directly targets both its GTP-bound ON- and GDP-bound OFF-states. Upon binding, the switch-II region adopts an open conformation in both structural states, suggesting that BBO-8520 forces KRAS G12C in the ON-state into a signaling-incompetent conformation [4].

In preclinical studies BBO-8520 showed potent antitumor activity in models resistant to KRAS G12C OFF-state inhibitors, including tumors with KRAS G12C amplification and receptor tyrosine kinase overexpression, highlighting its potential to overcome acquired resistance. NCT06343402 is a first-in-human phase 1 clinical trial to evaluate the safety and preliminary antitumor activity of BBO-8520 as a single agent and in combination with pembrolizumab and BBO-10203 in subjects with locally advanced and unresectable or metastatic NSCLC with a KRAS G12C mutation [4].

FMC-376 is another selective, orally bioavailable covalent inhibitor that targets both the ON- and OFF-states of KRAS G12C and has been shown to overcome resistance to first-generation inhibitors in preclinical models. It has demonstrated efficacy in patient-derived xenograft models of NSCLC, PDAC, and CRC, and is currently being evaluated in a phase 1/2 clinical trial (NCT06244771) [4].

#### 2.1.4. KRAS G12V Inhibitors

KRAS G12V represents the second most prevalent KRAS alteration overall: it is frequently detected in several pancreatic, lung, and colorectal malignancies. This variant differs from previously discussed KRAS alteration for two principal reasons. First, the glycine-to-valine substitution at codon 12 of the KRAS gene residue is targeted by small molecules through non-covalent interactions, rather than covalent binding. These agents require the formation of a ternary complex with CypA to achieve effective inhibition. Second, intrinsic GTP hydrolysis—from the active GTP-bound state to the inactive GDP-bound state—occurs more slowly in KRAS G12V, resulting in prolonged maintenance of the active (‘ON’) conformation and sustained oncogenic signaling.

For these reasons, there is an urgent unmet need to advance therapeutic strategies specifically directed against KRAS G12V-driven malignancies [4].

RMC-5127 is a potent, orally bioavailable, and selective non-covalent inhibitor that targets the active, GTP-bound form of KRAS G12V through a tri-complex mechanism. By forming a stable tri-complex with KRAS G12V in its GTP-bound ON-state and CypA, RMC-5127 induces the rapid disruption of downstream effector interactions via steric occlusion, effectively suppressing downstream signaling [4].

RMC-5127 has been evaluated as a therapeutic strategy in this context. The compound induces rapid suppression of ERK-mediated signal transduction, leading to cell-cycle arrest across multiple tumor cell lines, as well as in murine xenograft models. Notably, RMC-5127 has demonstrated measurable central nervous system penetration and favorable oral bioavailability [4].

A phase 1 clinical trial is currently underway (NCT07349537) to evaluate the safety, tolerability, PK, and preliminary antitumor activity of the drug as a monotherapy and in combination with either daraxonrasib or cetuximab in adults with KRAS G12V-mutant solid tumors [29].

#### 2.1.5. KRAS G12D Inhibitors

As already described in detail, irreversible KRAS G12C inhibitors have been approved for both NSCLC and mCRC, but no similar therapies are available for the KRAS G12D mutation, which is the predominant mutation subtype.

The G12D mutation results in intermediate intrinsic GTPase activity, a significantly impaired GDP-stimulated hydrolysis, and a comparative predisposition to activate the PI3K/AKT pathway rather than the MAPK/ERK pathway in the KRAS G12C mutant.

G12D introduces a carboxylate-containing aspartic acid residue that has low reactivity and high abundance on protein surfaces compared to the cysteine that replaces glycine in the G12C mutation, thus making it difficult to design irreversible inhibitors with sufficient selectivity and potency while maintaining single oral administration [30,31].

The success of KRAS G12C inhibitors stimulated further research targeting additional KRAS isoforms, particularly KRAS G12D, which is highly prevalent in PDAC.

In 2021, using a structure-based approach, Mirati et al. developed a potent and selective non-covalent inhibitor of KRAS G12D called MRTX1133, which binds to the inactive form of KRAS G12D (GDP-bound KRAS) with approximately 700-fold higher selectivity than to wild-type KRAS [32]. Furthermore, it is able to inhibit the binding of a peptide from the RAF-RAS binding domain to the active form of KRAS G12D (switch-II pocket).

The pharmacodynamic characterization of MRTX1133 was largely derived from in vivo studies. Notably, the compound demonstrated greater potency in three-dimensional organoid cultures compared to two-dimensional monolayer systems, suggesting a potential role in anchorage-dependent tumor growth. Mechanistically, MRTX1133 significantly reduced pERK1/2 and pS6 signaling.

Antitumor activity was observed in xenograft models derived from the human HPAC cell line, with significant tumor regression—occasionally complete—at a dose of 30 mg/kg administered twice daily. The most pronounced responses were observed in pancreatic cancer models. Importantly, MRTX1133 also appeared to modulate the tumor microenvironment. In immunodeficient murine models, the antitumor response was attenuated compared to immunocompetent models, suggesting a role for immune cell recruitment, particularly macrophages and lymphocytes, within the peritumoral niche [33].

Given the promising results obtained by MRTX1133 in preclinical studies [34], the drug was authorized by the FDA in October 2021 as an investigational new drug for a phase I/II clinical study (NCT05737706) intended for patients with advanced solid tumors (PDAC, NSCLC, CRC and others) with KRAS G12D mutation. However, although MRTX1133 pioneered this class of drugs, it does not appear destined for approval. Indeed, the data promised by Mirati et al. were never made available, and according to clinicaltrials.gov [35] the phase I/II study was quietly halted after the completion of phase I due to variable and suboptimal PK data [36]. Nevertheless, confidence in KRAS has not wavered.

RNK08954 is another non-covalent inhibitor of KRAS G12D. The compound preferentially binds the inactive GDP-bound state and exhibits high selectivity for KRAS G12D over other KRAS isoforms.

Preclinical studies were conducted both in vitro, using human pancreatic adenocarcinoma cell lines, as well as in murine cell models. RNK08954 induced significant inhibition of ERK1/2 and AKT signaling in a time- and dose-dependent manner across all tested doses (10, 30, and 100 mg/kg). The potency and overall activity of RNK08954 were reported to be substantially greater than those of MRTX1133.

In vivo studies, including GP2d xenograft models, demonstrated favorable PK properties characterized by a large volume of distribution, prolonged half-life, and high bioavailability. No significant changes in body weight were observed, consistent with favorable tissue selectivity and drug distribution. Single-cell RNA sequencing analyses further revealed substantial modulation of tumor-infiltrating lymphocytes and macrophages, particularly at the 100 mg/kg dose, findings that were corroborated by flow cytometric analysis. These observations provided a strong rationale for combination strategies with anti–PD-1 antibodies [37].

Preliminary results from an open-label Phase 1/2 study (NCT06667544) have shown encouraging clinical activity with oral RNK08954, particularly in patients with PDAC. Furthermore, the treatment was well tolerated (the main TRAEs of grade ≥ 3 included diarrhea (5%), vomiting (2%) and decreased appetite (5%)). A Phase 1B study in patients with PDAC is currently underway [38].

HRS-4642 is a non-covalent inhibitor with high selectivity and potency against KRAS G12D. It binds to both GDP- and GTP-bound KRAS G12D. Specifically, it prevents the interaction between GDP-bound KRAS G12D and SOS1, a guanine nucleotide-exchange factor responsible for KRAS activation. The binding of HRS-4642 to KRAS G12D, with an equilibrium dissociation constant (KD) value of 0.083 nM, was shown to be approximately 21-fold lower than that of KRAS G12C and 17-fold lower than that of wild-type KRAS protein, with a lower KD value indicating higher affinity [39].

A phase I study (NCT05533463) was initiated in 2022 in patients with advanced solid tumors harboring the KRAS G12D mutation to evaluate the PK, safety, and tolerability of HRS-4642 [40]. In 2023, Zhou and his colleagues presented the preliminary results of the phase 1 trial. Patients treated with HRS-4642 intravenously at doses of 15, 50, 100, 200, and 300 mg once weekly in 21-day cycles experienced no dose-limiting toxicities (DLTs), and the maximum tolerated dose (MTD) was not reached. Six patients (33.3%) experienced grade ≥ 3 TRAEs, including hypercholesterolemia (16.7%), increased lipase (11.1%), and anemia (11.1%), grade 2 increased ALT, and grade 1 increased AST. No patients discontinued treatment or died due to TRAEs. Exposure to HRS-4642 was approximately dose-proportional with a half-life of approximately 40 h [41].

A few years later, Xiong et al. showed that HRS-4642 administered intravenously to 84 patients (38 with NSCLC and 24 with PDAC) once weekly (QW: 200, 300, 400, or 500 mg), every two weeks (Q2W: 800 or 1200 mg), or every three weeks (Q3W: 1200 mg) also resulted in no DLT in 18 patients, and the MTD was not reached. Grade ≥ 3 TRAEs were reported in 20 of these patients (23.8%), the most common of which were hypertriglyceridemia, decreased neutrophil count, and hypercholesterolemia (three patients each [3.6%]). One patient (1.2%) discontinued treatment due to TRAEs. One treatment-related death occurred (1.2%). The terminal elimination half-life of HRS-4642 was approximately 3 days [42].

One strategy currently undergoing clinical evaluation is that of Weller et al. [43], who exploited the protein interface between RAS and CypA to catalyze irreversible inhibition of aspartate in mutant RAS. Among the resulting inhibitors is zoldonrasib, also known as RMC-9805. Zoldonrasib creates a ligand-mediated non-covalent protein–protein interaction between CypA and the active, GTP-bound state of RAS G12D isoforms (tri-complex inhibitor). Upon complex formation, a three-dimensional conformational change in the oncogenic protein occurs, leading to downstream blockade of cellular signaling pathways [44]. The subsequent covalent modification of the mutant Asp12 residue confers selectivity over wild-type RAS.

Zoldonrasib is currently under clinical investigation and has a corresponding preclinical analog, RMC-9945. The latter has been evaluated primarily in xenograft models of pancreatic adenocarcinoma, gastric adenocarcinoma, and NSCLC, demonstrating noteworthy and substantial response rates. In CRC xenograft models, responses were observed; however, the therapeutic benefit was comparatively modest. RMC-9945 is chemically stable, to the extent that a single daily administration in murine models is sufficient to maintain therapeutic efficacy [45].

Oral administration of zoldonrasib is currently undergoing clinical evaluation (multicenter, open-label phase 1/1b study—Sponsor Revolution Medicines, Inc-NCT06040541) in subjects with KRAS G12D-mutant solid tumors, who had previously received standard treatment [46]. Preliminary PK and safety data are available on the use of oral zoldonrasib in 90 patients with NSCLC treated with 1200 mg QD. During the dose escalation phase, no DLTs were reported at any dose or regimen, and the MTD of zoldonrasib was not reached. Unlike previous intravenously administered inhibitors, no grade 4 or 5 TRAEs or serious AEs were observed. Therefore, 1200 mg QD has been identified as the recommended dose for phase II. The most common TRAEs experienced with zoldonrasib (≥10% of patients) were nausea (39%), diarrhea (24%), vomiting (18%), and rash (12%). Only two TRAEs were grade 3 in severity: diarrhea in one patient and increased ALT in another. In 4% of patients, the dose was reduced, while in 9% treatment was discontinued due to a TRAE [47].

Preliminary PK and safety data on the use of oral zoldonrasib are also available in patients with PDAC. Again, no DLTs or grade 4 or 5 TRAEs were reported, and the MTD was not reached. Among patients who received a dose of 1200 mg QD, the most common TRAEs (≥10% of patients) were nausea (27%), diarrhea (20%), vomiting (15%), and rash (10%), all of which were grade 1 or 2 in severity. A grade 3 TRAE (ALT elevation) was observed in a patient with PDAC and a history of ALT elevation, biliary stent, and liver metastases. Four percent of patients had dose reductions due to TRAEs, but no patients discontinued treatment due to these [48].

INCB161734 is a next-generation, oral, selective and non-covalent ON/OFF-inhibitor of KRAS G12D, engineered to bind both the active and inactive forms of the mutant protein. Preclinical studies have demonstrated over 80-fold selectivity over wild-type KRAS, significant antitumor activity as monotherapy, and even greater tumor growth inhibition when combined with chemotherapy [49]. Within the field of KRAS G12D inhibition, INCB161734 warrants separate consideration. In fact, preclinical studies of this compound have been conducted using murine models and pancreatic tumor-derived cell lines. In these cellular systems, treatment with INCB161734 resulted in inhibition of cell-cycle progression, particularly at the S phase, associated with caspase-3/7 cleavage. This effect counteracts the hyperactivation of the ERK and PI3K signaling pathways that drive cellular proliferation. Furthermore, from a PK standpoint, the compound demonstrated excellent oral bioavailability in murine models [49].

In 2024 an open-label, multicenter phase I study (NCT06179160) was initiated to determine the safety and tolerability of INCB161734 as a single agent or in combination with other anticancer therapies in subjects with advanced or metastatic solid tumors harboring the KRAS G12D mutation [50]. As of 1 August 2025, 138 patients had been enrolled and received monotherapy with INCB161734 at doses ranging from 200 to 1600 mg QD. The results showed a manageable safety profile for the inhibitor. TRAEs occurring in ≥15% of patients, predominantly grade 1 and 2, included nausea (67%), diarrhea (58%), vomiting (50%), fatigue (22%), and increased lipase (17%). No patients experienced TRAEs leading to treatment discontinuation, and none experienced DLTs. MTD was not reached. Two of three patients treated with 1600 mg QD required a dose reduction, therefore the dose was not further increased [51].

Initial results on the use of INCB161734 in combination therapies in patients with PDAC and ≤1 prior treatment in the metastatic setting were also announced in 2026. The combination regimens included mFOLFIRINOX or GEMNabP. In patients who received INCB161734 600 mg in combination with GemNabP (*n* = 6) or mFOLFIRINOX (*n* = 4), no DLTs were observed, allowing for further dose escalation of INCB161734 with either chemotherapy. INCB161734, in combination with chemotherapy, showed steady-state exposure levels comparable to those of monotherapy, confirming an encouraging safety profile of the inhibitor even when administered concomitantly [52].

In the same year, another phase I/II study (NCT06500676) evaluated the safety/tolerability and PK of GFH375 monotherapy in patients with previously treated advanced solid tumors. GFH375 is another potent, highly selective oral inhibitor that binds both GDP- and GTP-bound KRAS G12D [53]. As of January 2025, 32 patients had been treated. No DLTs were observed at the doses tested [100 mg, 200 mg, 400 mg, 600 mg, 750 mg, 900 mg QD, and 300 mg BID]. Twenty-five percent of patients experienced at least one grade 3/4 TRAE and no grade 5 TRAEs, while 15.6% of patients experienced at least one serious AE. Two patients (6.3%) discontinued treatment due to TRAEs. No dose reductions occurred. The most common TRAEs were diarrhea (71.9%), vomiting (71.9%), and nausea (62.5%); all grade 1 or 2. GFH375 demonstrated good oral bioavailability with a Tmax of 2–4 h and a terminal half-life of 18.5–21.6 h [54]. As of August 2025, a total of 66 patients with PDAC had received GFH375 600 mg QD. No grade 5 TRAEs occurred. Grade 4 and grade 3 TRAEs occurred in 2% and 30% of patients, respectively. TRAEs resulted in dose reduction in 6% of patients and treatment discontinuation in 3%. The most common TRAEs (all grade G/≥G3) included diarrhea (56%/3%), decreased neutrophil count (49%/8%), vomiting (47%/1.5%), nausea (47%/0%), anemia (42%/8%), decreased white blood cell count (36%/2%), decreased appetite (33%/3%), hypoalbuminemia (33%/0%), decreased platelet count (29%/2%), and asthenia (26%/0%) [55].

Several other inhibitors targeting KRAS G12D are currently being evaluated in early-stage clinical trials, including TSN1611 [56], AZD0022 [57], and LY3962673 [58].

Another recent mechanism has been designed to target mutated KRAS G12D. It uses the cell’s ubiquitin-proteasome system to specifically degrade the oncogenic protein. As a proteolysis-inducing chimera (PROTAC), ASP3082 is the first drug to induce protein degradation rather than conventional inhibition [59]. Recently presented preliminary phase I data on ASP3082 in solid tumors with KRAS G12D mutations demonstrated a favorable safety profile in pretreated patients. The MTD was not reached. TRAEs occurred in 69.4% of patients (including five patients with grade 3 events). The TRAEs occurring in ≥5% of patients were fatigue (15.3%), infusion-related reactions (14.3%), pruritus (9.2%), nausea (7.1%), urticaria (7.1%), increased AST (7.1%), increased ALT (6.1%), and vomiting (5.1%). DLTs were observed at 450 mg (one case of grade 3 elevated ALT levels; one case of grade 3 elevated ALT/AST levels) and at 600 mg (one case of grade 3 elevated ALT levels) [60]. ASP3082 also showed a positive safety profile when taken in combination with mFOLFIRINOX, in patients with PDAC [61].

#### 2.1.6. Pan-RAS/Pan-KRAS Inhibitors

Pan-KRAS inhibitors provide new treatment options for KRAS mutant patients.

The Pan-RAS inhibitor that targets all mutant and wild-type subtypes has a theoretical advantage over targeting individual mutant alleles alone. Firstly, the relative rarity of certain KRAS alleles (such as Q61, G13X, etc.) makes it impractical to generate specific inhibitors for each point mutation, thus broadening the therapeutic potential of pan-RAS/KRAS drugs. Secondly, pan-targeted drugs may block the compensatory activation of the wild-type RAS subtype. Thirdly, pan-RAS/KRAS drugs may prevent the emergence of at least one acquired resistance pattern (targeted mutations within KRAS), and thus can also be used for treatment after directly targeting specific alleles [19].

Daraxonrasib (RMC-6236) is a RAS MULTI (ON) trimeric complex inhibitor which functions by forming a trimeric complex involving the active RAS protein and CypA. Importantly, it demonstrates activity against mutant and wild-type KRAS, NRAS, and HRAS.

Daraxonrasib has been evaluated in preclinical studies using NSCLC, PDAC, and CRC cell lines, as well as in murine xenograft models. The compound exhibited significant antitumor activity at doses as low as 10 mg/kg and demonstrated adequate central nervous system penetration. Moreover, such studies have shown its effectiveness in tumors with diverse RAS genotypes, including cancer models that have developed resistance to KRAS G12C OFF-inhibitors due to factors like secondary NRAS mutations, KRAS amplification, and RTK amplification [19].

Importantly, by selectively targeting the active GTP-bound state of KRAS, daraxonrasib may overcome resistance mechanisms associated with GDP-state inhibitors. A distinctive feature of this agent is its ability to promote in vivo immune activation, thereby providing a rationale for combination strategies with PD-1/PD-L1 immune checkpoint inhibitors. Objective tumor responses were observed as early as the second administration [62].

The first clinical efficacy data for this compound were presented at the 2023 ESMO conference (NCT05379985). Patients with previously treated, advanced KRAS G12-mutant solid tumors were enrolled at escalating doses (10 mg to 160 mg QD) of daraxonrasib. Additional patients with PDAC or NSCLC were enrolled at dose levels that cleared dose-limiting toxicity evaluation. TRAEs occurring in ≥10% of patients were rash (52%), diarrhea (21%), nausea (21%), and vomiting (15%). The only grade ≥ 3 TRAE was in a patient with PDAC who had a grade 4 large intestine perforation at the site of an invasive tumor that reduced in size on treatment [63].

BI-2493 and BI-2865 exhibit selective binding to KRAS-GDP. Their mechanism of action involves blocking nucleotide exchange, thereby preventing the activation of both wild-type and mutant KRAS while sparing other RAS family proteins. This selectivity is key to minimizing damage to normal cells. Both inhibitors have demonstrated promising tumor suppression effects in both in vitro and in vivo studies [19].

BI-1701963 is a small-molecule inhibitor of SOS1. By binding to SOS1, the compound disrupts its interaction with KRAS, thereby preventing GDP/GTP exchange and inhibiting the KRAS conversion to the ON-state. For this reason, BI-1701963 is classified as an indirect KRAS inhibitor [64]. The molecule was entered into a phase I clinical trial (NCT04111458) whose objective was to evaluate BI 1701963 as monotherapy or in combination with trametinib in adults with KRAS-mutated solid tumors [65].

AMG 410, in contrast, is a dual GTP(ON)- and GDP(OFF)-state inhibitor (KD(GDP-state) = 1 nM; KD(GTP-state) = 22 nM), enabling blockade of KRAS signaling in a cycling state-independent manner, while also allowing AMG 410 to block proliferation in wild-type KRAS-amplified tumor cells [64]. AMG 410 shows potent antiproliferative activity in KRAS-mutant cells (median IC50 = 12 nM). Importantly, AMG 410 is highly specific for KRAS, demonstrating greater than 100-fold selectivity against both HRAS and NRAS, differentiating AMG 410 from pan-RAS inhibitors through its ability to avoid antiproliferative effects in non-KRAS-transformed cells (median IC50 > 5 µM) [66].

AMG 410 demonstrates strong preclinical efficacy, robustly lowing phosphorylated ERK levels throughout dosing cycles and achieving tumor stasis or regression across a broad set of colorectal, pancreatic, and lung cancer cell line-derived xenograft and patient-derived xenograft models harboring a diverse set of KRAS mutant alleles including G12D, G12V, G12C, and G13D. AMG 410 also shows enhanced in vivo efficacy in combination with other targeted therapies or immunotherapy and good preclinical tolerability, highlighting the potential of an HRAS- and NRAS-sparing pan-KRAS inhibitor to show improved efficacy and clinical tolerability in the combination setting [66].

Based on a promising nonclinical safety and efficacy profile, a first-in-human study with AMG 410 in a range of solid tumor indications (CRC, PDAC, NSCLC) is now ongoing (NCT07094113).

ERAS-0015 is a pan-RAS molecular glue with best-in-class potential designed to target RAS in the GTP-bound state and shut down cellular signaling mediated by both mutant and wild-type RAS. ERAS-0015 exhibited 3–7-fold more potent inhibition of cellular proliferation relative to daraxonrasib, a RAS molecular glue comparator. ERAS-0015’s roughly 8–20-fold higher binding affinity to CypA is thought to contribute to its higher in vitro cellular potency as well as favorable PK properties resulting in a longer residence time and greater exposure in tumor tissues as compared to daraxonrasib [67].

**Figure 2:**
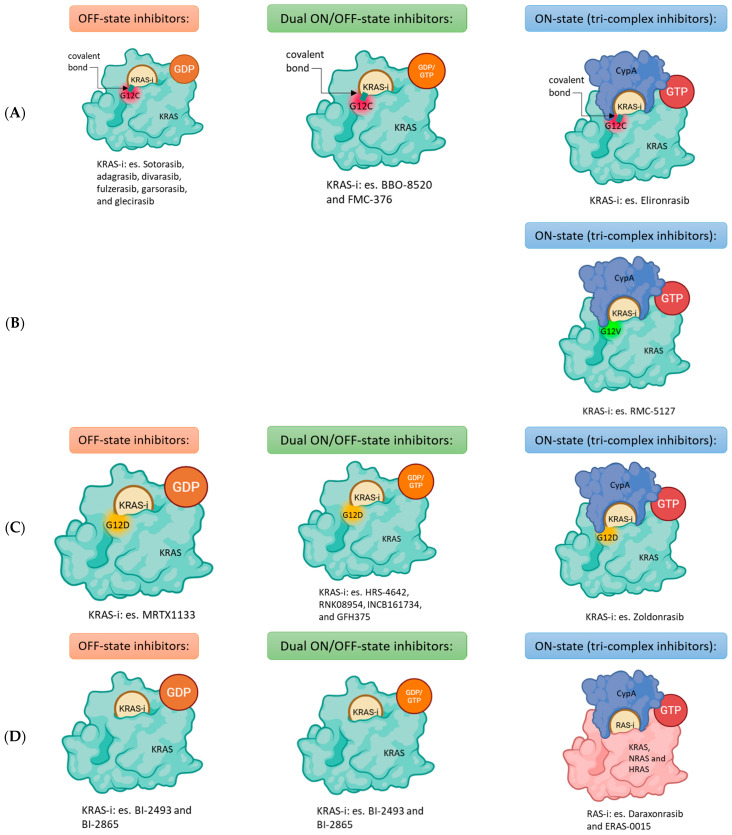
Mechanism of action of RAS inhibitors. Schematic overview of different mechanisms of action of KRAS OFF-, ON- and dual ON/OFF-state G12C inhibitors (**A**), KRAS ON-state G12V inhibitors (**B**), KRAS OFF-, ON- and dual ON/OFF-state G12D inhibitors (**C**) and Pan-RAS/Pan-KRAS OFF-, ON- and dual ON/OFF-state inhibitors (**D**). KRAS OFF-state inhibitors bind to the mutant KRAS, locking the protein in an inactive GDP-bound OFF-state. KRAS ON-state inhibitors bind to the cyclophilin A (CypA) and to the mutant RAS GTP-bound ON proteins, with the formation of an irreversible inhibitory tri-complex in which CypA sterically occludes the RAS effector binding face. KRAS dual ON/OFF-state inhibitors bind to the mutant KRAS and directly target both its GTP-bound ON- and GDP-bound OFF-states. This dual-state targeting offers a significant opportunity to overcome the resistance mechanism. Created in BioRender. Filippelli, A. (2026), https://biorender.com/r2gmi0h.

**Figure 3:**
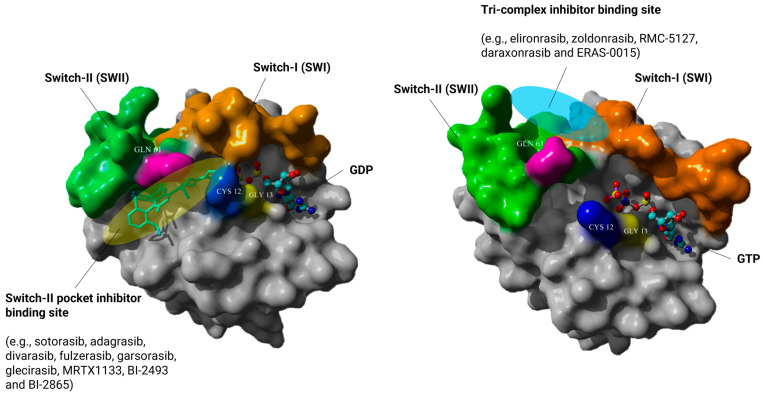
Structure of KRAS GDP-bound OFF-state (**left**) and KRAS GTP-bound ON-state (**right**). The figure shows switch-I (aa 30–38), switch-II (aa 60–76) and common oncogenic mutation sites (CYS12 [G12], GLY 13 [G13], and GLN 61 [Q61]). KRAS cycles between the inactive GDP-bound OFF- and active GTP-bound ON-states: switch-II pocket inhibitors (e.g., sotorasib, adagrasib, divarasib, fulzerasib, garsorasib, glecirasib, MRTX1133, BI-2493 and BI-2865) target GDP-bound KRAS, while tri-complex inhibitors (e.g., elironrasib, zoldonrasib, RMC-5127, daraxonrasib and ERAS-0015) engage the binding site between switch-I and -II to target GTP-bound KRAS. Created with YASARA (Yet Another Scientific Artificial Reality Application) [68].

Figure 2 resumes the mechanisms of action of different classes of RAS inhibitors while Figure 3 shows the structure of GTP/GDP bound KRAS.

For further details on the pharmacological properties and tolerability of RAS inhibitors currently under development, please refer to Table 2.

## 3. Clinical Development of KRAS Inhibitors: From Selective Inhibitors to KRAS on Inhibitors

As mentioned above, the distribution of KRAS mutations varies substantially across tumor types and has directly influenced the clinical development strategy of KRAS-targeted therapies [69].

Recent reviews have highlighted how the biological heterogeneity of KRAS-mutant tumors, the emergence of adaptive resistance mechanisms, and tumor-specific signaling dependencies continue to shape the development of KRAS-directed therapeutic strategies across different cancer types [3,70].

The first proof of clinical activity of KRAS G12C inhibition was demonstrated in NSCLC, where this mutation is most prevalent and clinically relevant. Sotorasib was evaluated in the phase I/II CodeBreaK 100 basket trial, which enrolled patients with advanced KRAS G12C-mutated solid tumors, including NSCLC, CRC, and pancreatic cancers. The largest and most clinically relevant cohort consisted of patients with NSCLC. In the phase II expansion cohort, the primary endpoint was objective response assessed by independent central review, with secondary endpoints including duration of response, disease control, progression-free survival (PFS), overall survival (OS), and safety.

Among 126 previously treated patients, objective responses were observed in 37.1% of cases, including 3.2% complete responses and 33.9% partial responses. The median duration of response was 11.1 months, with an mPFS of 6.8 months and a median OS (mOS) of 12.5 months [71]. Sotorasib demonstrated a manageable safety profile, with treatment-related adverse events occurring in 69.8% of patients and grade 3 or higher events in approximately 20%. The most common adverse events included diarrhea, nausea, transaminase elevations, and fatigue, with hepatotoxicity and gastrointestinal toxicity representing the most clinically relevant toxicities. Importantly, responses were observed across PD-L1 expression levels and were independent of tumor mutational burden and STK11/TP53 status, although numerically lower response rates were reported in tumors harboring KEAP1 mutations [71].

These findings were subsequently confirmed in the phase III CodeBreaK 200 trial, in which sotorasib significantly improved ORR (28.1% vs. 13.2%) and PFS (median 5.6 vs. 4.5 months; HR 0.66, 95% CI 0.51–0.86; *p* = 0.0017) compared with docetaxel, along with higher disease control rates (82.5% vs. 60.3%). However, no overall survival benefit was observed (median 10.6 vs. 11.3 months; HR 1.01, 95% CI 0.77–1.33). This lack of overall survival benefit may be partly explained by the high rate of crossover to KRAS G12C inhibitors in the control arm. In addition, several limitations of the trial design have been highlighted, including the use of docetaxel monotherapy as a comparator, which may not fully reflect all therapeutic options available in certain clinical settings [4]. In parallel, a randomized phase III trial is currently evaluating sotorasib versus pembrolizumab in combination with platinum-based doublet chemotherapy as a first-line treatment for patients with advanced PD-L1-negative NSCLC, with primary completion anticipated in 2026 (NCT05920356) [72].

Adagrasib, another mutant-selective covalent KRAS G12C OFF-state inhibitor, shares a similar mechanism of action with sotorasib but is characterized by a notably longer half-life [14]. In the phase I/II KRYSTAL-1 study, adagrasib demonstrated clinically meaningful activity, achieving an ORR of 43%, with a median DOR, PFS, and OS of 8.5, 6.5, and 12.6 months, respectively [73].

Notably, in a dedicated cohort of patients with untreated brain metastases, adagrasib showed intracranial activity, with an objective response rate of 42% and durable central nervous system disease control, albeit in a limited patient population [74].

Adagrasib was associated with a manageable safety profile, with predominantly gastrointestinal and hepatic adverse events. Grade ≥ 3 treatment-related adverse events occurred in approximately 40–45% of patients, most commonly elevated transaminases and gastrointestinal toxicity, and were generally manageable with dose modification. Responses were observed across major genomic subgroups, including tumors harboring STK11, TP53, and CDKN2A alterations. Although activity was maintained in STK11-mutant disease, numerically lower response rates were reported in tumors with KEAP1 co-mutations, particularly in the STK11–wild-type/KEAP1-mutant subgroup, while responses were consistent across PD-L1 expression levels [73].

Building on the phase II results of KRYSTAL-1, the randomized phase III KRYSTAL-12 trial (NCT04685135), patients with previously treated advanced KRAS G12C-mutant NSCLC were randomized to receive adagrasib (600 mg orally BID) or docetaxel (75 mg/m^2^ IV every three weeks). Preliminary results, reported in abstract form, demonstrated a significant improvement in progression-free survival with adagrasib compared with docetaxel (median 5.49 vs. 3.84 months; HR 0.58, 95% CI 0.45–0.76; *p* < 0.0001), along with a higher ORR (31.9% vs. 9.2%; OR 4.68, 95% CI 2.56–8.56; *p* < 0.0001). Treatment-related adverse events were reported in 94.0% of patients receiving adagrasib and 86.4% of those receiving docetaxel, with comparable rates of grade 3–4 events between the two groups (47.0% vs. 45.7%) [75].

These findings further support the clinical activity of KRAS G12C inhibition in previously treated NSCLC, while also highlighting the need for more effective and durable therapeutic strategies. In this context, several next-generation KRAS G12C inhibitors are currently under investigation.

The randomized phase III KRASCENDO-1 trial is currently evaluating the efficacy of divarasib compared with sotorasib or adagrasib in patients with previously treated advanced NSCLC, with primary completion expected in 2026 (NCT06497556) [76].

Beyond allele-specific KRAS inhibitors, broader RAS-targeting strategies are emerging as a promising therapeutic approach. Daraxonrasib recently demonstrated significant clinical benefit in the phase III RASolute 302 trial. Among patients with previously treated metastatic PDAC, daraxonrasib significantly improved OS compared with investigator’s-choice chemotherapy (median OS, 13.2 vs. 6.6 months; HR 0.40; *p* < 0.001) and prolonged PFS (median PFS, 7.3 vs. 3.5 months; HR 0.45; *p* < 0.001) in patients harboring RAS G12 mutations. These findings support the potential of RAS(ON) inhibition as a next-generation strategy for targeting a broader spectrum of RAS-driven malignancies [77].

In addition, several other next-generation covalent KRAS G12C inhibitors, including glecirasib, fulzerasib, and garsorasib, are currently under clinical development, with emerging data summarized in Table 3.

The growing interest in combination strategies is supported by evidence that oncogenic KRAS signaling contributes to the establishment of an immunosuppressive tumor microenvironment and promotes adaptive signaling pathways that may limit the durability of response to KRAS-targeted monotherapy [70].

The phase Ib CodeBreaK 101 study evaluating sotorasib in combination with pembrolizumab or atezolizumab was discontinued due to significant hepatotoxicity [78]. In contrast, more encouraging results have been reported from the phase II KRYSTAL-7 trial, in which adagrasib combined with pembrolizumab demonstrated an objective response rate of 61% and an mPFS survival of 27.7 months in treatment-naïve patients with advanced NSCLC and PD-L1 expression ≥ 50%, with a manageable safety profile [79,80].

Several randomized phase III trials are currently ongoing to evaluate KRAS G12C inhibitors in combination with pembrolizumab with or without chemotherapy as first-line therapy for advanced NSCLC, including adagrasib (NCT04613596, NCT06875310), (NCT06793215). Primary completion for most of these studies is anticipated between 2028 and 2029.

Collectively, these data highlight the rapid evolution of KRAS-targeted therapies, with ongoing efforts focused on improving the depth and durability of responses through next-generation inhibitors and combination strategies.

In contrast to NSCLC, the clinical activity of KRAS G12C OFF-state inhibitors as monotherapy in metastatic CRC remains modest. In the phase II CodeBreaK 100 trial, sotorasib achieved an ORR of 9.7% and a median PFS (mPFS) of 4.0 months in the CRC cohort [81]. Although cross-trial comparisons indicate somewhat higher response rates with adagrasib (23% in KRYSTAL-1) [82], divarasib (29.1% in NCT04449874) [83], and other emerging KRAS G12C inhibitors, overall clinical benefit remains limited, with mPFS generally ranging from approximately 5.6 to 7.8 months [82].

Preclinical evidence suggests that the limited efficacy of KRAS G12C inhibitors in CRC is driven, at least in part, by rapid reactivation of the RAS/MAPK pathway through adaptive upregulation of receptor tyrosine kinases such as EGFR, leading to bypass signaling via wild-type RAS. This adaptive feedback signaling is recognized as an important mechanism limiting the efficacy of KRAS G12C inhibitor monotherapy in CRC and provides a strong biological rationale for vertical pathway inhibition strategies [70]. These findings, together with prior clinical success of vertical pathway inhibition in BRAF V600E-mutant CRC, provided the rationale for combining KRAS G12C inhibitors with anti-EGFR therapies in metastatic CRC [4].

In parallel, the randomized phase III KRYSTAL-10 trial has completed enrollment and is evaluating adagrasib in combination with cetuximab versus standard-of-care therapy as second-line therapy in advanced CRC (NCT04793958) [84]. In addition, early-phase clinical results with divarasib plus cetuximab have shown particularly encouraging activity, with an objective response rate of 62.5% reported in a phase Ib trial (NCT04449874), including responses in patients who had previously received KRAS G12C inhibitors [85].

The first randomized evidence supporting the combination of KRAS G12C inhibitors with anti-EGFR therapy was provided by the phase III CodeBreaK 300 trial. In this study, sotorasib (960 mg QD) combined with panitumumab significantly improved progression-free survival compared with standard-of-care therapies (trifluridine-tipiracil or regorafenib), with a mPFS of 5.6 versus 2.0 months and an objective response rate of 30.2%, ultimately leading to FDA approval in chemotherapy-refractory metastatic CRC. Although no statistically significant overall survival benefit was observed—likely due to limited statistical power—a favorable trend in OS was noted in the combination arm, supporting its clinical relevance [86]. Additional evidence from the phase 1b CodeBreaK 101 trial further supports this strategy, with sotorasib plus panitumumab administered alongside FOLFIRI in the first-line setting demonstrating a high level of antitumor activity. In this cohort, an objective response rate of 78% was observed, with tumor reduction reported in all evaluable patients, alongside a manageable safety profile [87].

Several phase III trials are currently underway to assess KRAS G12C-targeted strategies in the first-line setting for advanced CRC, including sotorasib in combination with panitumumab and chemotherapy (NCT06252649) [88] as well as MK-1084 combined with cetuximab and chemotherapy (NCT06997497). Taken together, these ongoing studies indicate that mutant-selective KRAS G12C OFF-state inhibitors may soon move into earlier lines of treatment and potentially expand into (neo)adjuvant therapeutic settings in CRC.

While KRAS G12C OFF-state inhibitors are currently approved only for NSCLC and CRC—where this mutation is most prevalent—emerging clinical evidence indicates potential activity across a broader range of tumor types. In the CodeBreaK 100 study, a subgroup analysis showed an objective response rate of 14.3% in patients with KRAS G12C-mutant malignancies outside NSCLC and CRC [81]. In heavily pretreated patients with advanced PDAC, a population with particularly poor prognosis and limited therapeutic options, sotorasib achieved an ORR of 21% with a mPFS of 4.0 months [89].

In cross-trial comparisons, adagrasib appeared to provide somewhat higher activity, with an ORR of 35.1% across multiple KRAS G12C-mutant tumor types, including PDAC (ORR 33.3%, mPFS 5.4 months), biliary tract cancers (ORR 41.7%, mPFS 8.6 months), and gynecologic malignancies (ORR 57.1%, mPFS 8.1 months) [90].

Furthermore, preliminary signals of efficacy have been reported with several next-generation KRAS G12C inhibitors—such as divarasib, glecirasib, and fulzerasib—in PDAC and other malignancies, as summarized in Table 4.

## 4. Resistance to KRAS Inhibitors and Combination Strategies

As the above-presented data eloquently show, there is great preclinical and clinical investigational interest in the study and development of KRAS inhibitors and these agents have already started to impact our clinical practice, providing robust safety, tolerability and efficacy results in the treatment of advanced NSCLC and CRC and encouraging results for PDAC. However, mechanisms of resistance are frequent and contribute to significantly dampen long-lasting survival benefits [91,92]. Broadly speaking, resistance mechanisms can be categorized as primary/intrinsic (when cancer cells are innately resistant to a given treatment, before exposure) or secondary/acquired (arising after treatment exposure), and can be further divided into on-target (with alterations developing in the target oncogene) and off-target (with alterations not developing in the target oncogene) [93].

While KRAS G12V selective inhibitors are still early investigational agents, whose data do not yet provide significant insight into their specific resistance mechanisms [94,95,96,97,98], the same does not apply to KRAS G12D inhibitors, which provide a limited yet solid body of preclinical evidence in this respect, while KRAS G12C inhibitors represent our main source of large clinical understanding in this field.

With reference to resistance mechanisms arising after KRAS G12D inhibitor treatment, Dilly et coll. derived cell lines and organoids from PDAC-affected, KRAS G12D-mutated patients and treated them with the KRAS G12D inhibitor MRTX1133, observing that PI3K-AKT-mTOR signaling and EMT (Epithelial-to-Mesenchymal Transition) were strongly linked to therapy resistance; in addition, resistant cell lines presented a distinct CNV (Copy Number Variation) profile with gains of EGFR, MET, BRAF, ETV1, ZEB1 and TWIST1, alongside with transcription signatures associated with cell proliferation, EMT, MYC. Similarly, in KPC (KrasLSL−G12D/+; Trp53LSL − R172H/+; p48-Cre) mouse models, therapy resistance was linked to EMT and to KRAS, YAP1, MYC, ABCB1A/B and CDK6 gene amplification [99]. In the same line, Maruyama et coll. analyzed intrinsic resistance mechanisms in patient-derived KRAS-G12D-mutated CRC cell lines, reporting MAPK and PI3K pathway activation as the main mechanisms involved [100]. In another extremely thought-provoking, well-conducted and epigenetic-oriented study, Principe et coll. generated MRTX1133-resistant human (PANC1, ASPC1, and CD18) and murine (KPC-2138 and KPC-3213) PDAC cell lines via incubation in increasing concentrations of MRTX1133. RNA sequencing revealed a switch towards histone acetylation, linked to the activation of anti-apoptosis FOSL1 signaling: treatment with EP300 (a histone acetyltransferase inhibitor) prevented this switch and thus inhibited FOSL1, as confirmed by RNA sequencing and by the similar outcome obtained with siFOSL1. Building on these findings, Principe et coll. demonstrated similar results employing BET (Bromodomain and Extra-Terminal domain) inhibitors (JQ1 or OTX-015 or ABBV-744 or trotabresib, which are currently under clinical investigation, unlike EP300 or FOSL1-inhibitors) on the same cell lines. Furthermore, they also proved that combining MRTX1133 with JQ1 significantly increases the OS in the 2138 K and 1245 K KPC-derived mice models [101].

With reference to mechanisms of resistance to KRAS G12C inhibitors, a well-rounded and ever-growing body of preclinical and clinical evidence has as of now established the main intrinsic and acquired resistance mechanisms in this subset of patients. The more robust and extensive data also derives from preclinical models, but they mainly derive from sotorasib and adagrasib treatment experiences in advanced NSCLC and (in a numerically much less significant way) CRC and PDAC patients: in fact, we can now analyze the data coming from more than 200 KRAS G12C inhibitor-treated patients, collected from 10 different studies [102]. Co-occurring baseline genetic alterations seem to represent the major intrinsic resistance mechanism, with KEAP1, SMARCA4, and CDKN2A being the most frequent ones in advanced NSCLC and KRAS, NRAS, BRAF, TP53 mutations, EGFR amplifications and PI3K pathway mutations being the most frequent ones in advanced CRC and PDAC [103,104]. On the other hand, with respect to acquired resistance mechanisms to KRAS G12C inhibitors, secondary resistance mutations seem to represent the main subset, accounting for approximately 60% of all acquired resistance mechanisms; the same mutations are described for advanced NSCLC, CRC and PDAC patients, the main difference being that advanced CRC patients seem to present multiple secondary resistance mutations at once [105,106].

Among secondary resistance mutations, on-target KRAS alterations constitute the leading resistance mechanism and are mainly represented by mutations in the switch-II binding pocket, such as R68S, H95D/Q/R, and Y96C, which are especially dangerous given the fact they stop KRAS G12C inhibitor molecules from binding, KRAS mutations other than G12C (such as G12V and G12D) and KRAS amplifications [107,108,109].

Off-target alterations are also commonly found, with RTK-RAS-MAPK and PI3K pathway alterations constituting the most described ones [110,111]; downstream KRAS regulators mutations are also described, albeit less frequently, in the form of MYC amplifications or CNV gains and YAP/TAZ amplification or activation [112].

Last, but not least, non-genetic resistance mechanisms can also be found in patients exposed to KRAS G12C inhibitors; in this respect, as of today two major such mechanisms have been described: the switch towards a highly immunosuppressive TME and the EMT [113,114].

**Figure 4:**
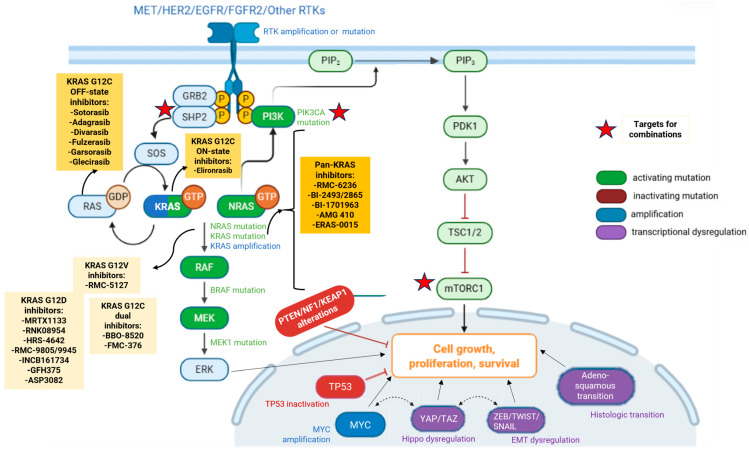
Molecular mechanisms of resistance to KRAS-targeted therapies and emerging therapeutic strategies in KRAS-mutant cancers. Genetic alterations associated with primary or acquired resistance include mutations in PIK3CA, NRAS, BRAF, and MEK1, as well as KRAS amplification. Additional mechanisms involve epithelial–mesenchymal transition (EMT), adenosquamous transdifferentiation, dysregulation of the Hippo/YAP–TAZ pathway, TP53 inactivation, and MYC amplification. The figure also summarizes KRAS-targeted therapeutic approaches, including mutant-selective inhibitors targeting KRAS G12C, G12D, and G12V variants, dual KRAS inhibitors, pan-KRAS inhibitors, and potential targets for combination therapies. Created in BioRender. Filippelli, A. (2026), https://biorender.com/r2gmi0h.

Figure 4 summarizes principal molecular mechanisms of resistance to KRAS-targeted therapies.

## 5. Conclusions

The clinical development of KRAS G12C inhibitors represented a fundamental shift that opened the way to a new class of drugs. However, RAS mutations are heterogeneous and have unique impacts on clinical outcomes, depending also on the tumor type and co-mutations. Multiple efforts are needed to decipher this heterogeneity and to implement diagnostic strategies able to correctly identify precise molecular alterations, in light of the specificity of each inhibitor.

## Figures and Tables

**Table 1:** Pharmacological and safety profile of sotorasib and adagrasib.

	Sotorasib [1,4,9,10,11]	Adagrasib [1,4,13,15,16]
**Posology and** **administration**	Recommended dose 960 mg OA QD; dose reduction to 480 mg QD then 240 mg QD for AEs; discontinuation if 240 mg QD is not tolerated.	Recommended dose: 600 mg OA BID; dose reduction to 400 mg BID then 600 mg QD for AEs; discontinuation if 600 mg QD is not tolerated.
**Pharmacokinetics**	Steady state: 22 days; T1/2: 5 h; Median Cmax: 1 h; Administration with a high-fat, high-calorie meal increases AUC 0–24 h by 25%.	Steady state: 8 days; T1/2: 23 h; Median Cmax: ~6 h; Administration with a high-fat, high-calorie meal does not affect pharmacokinetics.
**Distribution and** **Metabolism**	89% bound to plasma proteins; Vd: 211 L; Metabolized primarily by CYP3A4.	~98% bound to plasma proteins; Apparent Vd: 942 L; Metabolized primarily by CYP3A4.
**Excretion**	74% of dose recovered in feces (53% unchanged) and 6% in urine (1% unchanged). Apparent clearance: 26.2 L/h.	~75% of dose recovered in feces (14% unchanged) and 4.5% in urine (2% unchanged). Apparent clearance: 37 L/h.
**Special** **populations**	Age, race, sex, body weight, line of treatment, ECOG PS (0 or 1), mild/moderate renal impairment, or mild hepatic impairment do not significantly influence pharmacokinetics.	Age, sex, race, body weight, tumor burden, ECOG PS, mild-to-severe renal or hepatic impairment do not significantly influence pharmacokinetics.
**Drug** **interactions**	Avoid coadministration with: PPIs, H2 receptor antagonists, strong CYP3A4 inducers. Avoid coadministration with CYP3A4 substrates, for which minimal concentration changes may lead to therapeutic failures of the substrate, and P-gp substrates, for which minimal concentration changes may lead to serious toxicities.	Avoid coadministration with: strong CYP3A inducers or inhibitors, and sensitive CYP3A, CYP2C9, CYP2D6, or P-gp substrates. Avoid use with other products with a known potential to prolong the QTc interval.
**Most frequent adverse events**		
**Any grade** **(≥20%)**	Diarrhea, musculoskeletal pain, nausea, vomiting, fatigue, hepatotoxicity, cough and ILD.	Diarrhea, musculoskeletal pain, nausea, vomiting, fatigue, hepatotoxicity, renal impairment, dyspnea, edema, decreased appetite, cough, pneumonia, dizziness, constipation, abdominal pain and QTc prolonged.
**Laboratory** **abnormalities** **(≥25%)**	Decreased lymphocytes, decreased hemoglobin, increased AST, increased ALT, decreased calcium and increased alkaline phosphatase.	Decreased lymphocytes, increased AST, decreased sodium, decreased hemoglobin, increased creatinine, decreased albumin, increased ALT, increased lipase, decreased platelets, decreased magnesium and decreased potassium.
**Grade 3 or 4** **(≥5%)**	Hepatotoxicity, increased ALT, increased AST, musculoskeletal pain, pneumonia and diarrhea.	Pneumonia, hepatotoxicity, dyspnea, fatigue, musculoskeletal pain, renal impairment and prolonged QTc.
**Serious and fatal AEs**		
**Serious AEs**	Pneumonia, hepatotoxicity and diarrhea.	Pneumonia, dyspnea and renal impairment.
**Fatal AEs**	Pneumonia, respiratory failure, cardiac arrest, cardiac failure and gastric ulcer.	Pneumonia, respiratory failure, sudden death and cardiac failure.

Abbreviations: AE, adverse event; ALT, alanine aminotransferase; AST, aspartate aminotransferase; AUC, area under the concentration–time curve; BID, twice daily; Cmax, maximum plasma concentration; CYP, cytochrome P450; ECOG PS, Eastern Cooperative Oncology Group Performance Status; ILD, interstitial lung disease; OA, oral administration; P-gp, P-glycoprotein; PPIs, proton pump inhibitors; QD, once daily; QTc, corrected QT interval; T1/2, half-life; Vd, volume of distribution.

**Table 2:** Pharmacological and safety profile of KRAS inhibitors in clinical development.

Drug/ Company	Binding Type	Route of Administration	Clinical Trial Phases	Tumor Type	PK	DLTs	Recommended Dose	MTD	TRAEs or Serious AEs	Reference
**KRAS G12C inhibitors**										
**Divarasib** **(GDC-6036)/** Genentech (South San Francisco, CA, USA)	OFF-state	OA	phase 1	Advanced or metastatic solid tumors: NSCLC (*n* = 60), CRC (*n* = 55), and other solid tumors (*n* = 22)	Average T1/2: 17.6 h (±2.7 h); Mean Cmax: 657 nanograms per milliliter (±185); Mean AUC: 9130 nanograms x hours per milliliter (±3160)	NA	400 mg daily	Not reached	AEs (93% of patients). This included 11% grade 3 and 1% grade 4 AEs. Most common AEs in the NSCLC cohort: nausea (78%), vomiting (63%), diarrhea (60%), and fatigue (27%). No grade 5 AEs.	[17,18]
**Fulzerasib (IBI351)/** Innovent Biologics (Suzhou, Jiangsu, China)	OFF-state	OA	Phase 1a/1b	In phase 1a: NSCLC (*n* = 69), CRC (*n* = 6), and PDAC (*n* = 1); in phase 1b: NSCLC (*n* = 97), intestinal cancer (*n* = 1), melanoma (*n* = 1), and ovarian cancer (*n* = 1)	Tmax: ~2 h; T1/2: ~5 h after a single dose	Not observed	600 mg BID	NA	TEAEs (98.9% of patients), TRAEs (95.5% of patients); most commonly anemia (47.7%), increased ALT (33.0%), and increased AST (31.3%). Grade ≥ 3 TRAEs (36.4% of patients); most frequently increased GGT (10.2%) and anemia (6.8%); TEAEs leading to death (5.7% of patients); 2 deaths (1.1%) were suspected to be treatment-related.	[21]
**Garsorasib** **(D-1553)/** InventisBio (Shanghai, China)	OFF-state	OA	Phase 1	NSCLC (*n* = 79)	Tmax: 1–4 h after a single oral dose and 1–2 h after 600 mg BID. T1/2: approximately 3–6 h. Geometric mean Cmax on day 14: 12.5 mM. Cmin after 600 mg BID: 1.05 mM; Cmin after 600 mg QD: 0.20; mM; AUC0-24 after 600 mg BID: 127.5 h mM; AUC0-24 after 600 mg QD: 62.1 h mM.	Not observed	600 mg BID		TRAEs (94.9% of patients); most of which were grade 1 to 2; the most common (≥10% of patients); liver function abnormalities and Gl events. Grade 3 or 4 AEs (38.0% of patients); mainly liver function abnormalities and GI events.	[22]
**Glecirasib (JAB-21822)/** Jacobio Pharma (Beijing, China)	OFF-state	OA	Phase 1/2a	Advanced or metastatic solid tumor (NSCLC, CRC and PDAC)	Average Tmax: 2 h. T1/2: approximately 4.99–5.54 h	Not observed	800 mg daily		TRAEs (≥10% of patients): anemia (24.5%), total bilirubin increase (20.8%), direct bilirubin increase (15.1%), proteinuria (13.2%) and indirect bilirubin increase (11.3%).	[19,23]
**Elironrasib (RMC-6291)/** Revolution Medicines (Redwood City, CA, USA)	ON-state	OA	Phase 1	Advanced mutated solid tumor (including NSCLC and CRC)	Median Tmax: 1.0 h; Terminal T1/2: 1.8 h	NA	NA	NA	TRAEs (≥10% of patients); nausea (34%), diarrhea (30%), QTc prolongation (21%), vomiting and fatigue (15% each). Grade 3 TRAEs: QTc prolongation. No grade ≥ 3 hepatotoxicity TRAEs observed. No grade 4 or 5 TRAEs or TEAEs that led to treatment discontinuation.	[27]
**BBO-852/** BridgeBio Oncology Therapeutics (South San Francisco, CA, USA)	Dual state (ON/OFF)	OA	Phase 1	NSCLC	NA	NA	NA	NA	NA	
**FMC-376**/ Frontier Medicines Corporation (South San Francisco, CA, USA)	Dual state (ON/OFF)	OA	Phase 1/2	Advanced unresectable or metastatic solid tumors	NA	NA	NA	NA	NA	[4]
**KRAS G12V inhibitors**										
**RMC-5127**/ Revolution Medicines, Inc.	ON-state (tri-complex inhibitor)	OA	Phase 1/1b	NA	NA	NA	NA	NA	NA	[4]
**KRAS G12D inhibitors**										
**MRTX1133/** Mirati (San Diego, CA, USA)/ Bristol Myers Squibb (Princeton, NJ, USA)	OFF-state	IV	Phase 1/2	NA	NA	NA	NA	NA	NA	
**HRS-4642/** Hengrui Pharma (Lianyungang, Jiangsu Province, China)	Dual state (ON/OFF)	IV	Phase 1	Lung adenocarcinoma (*n* = 10), CRC (*n* = 5), appendiceal mucinous adenocarcinoma, ovarian cancer, and pancreatic cancer (*n* = 1 each)	T1/2: 40 h	Not observed	NA	Not reached	Serious AEs; Grade 2: increased ALT (5.5%), Grade 1: increased AST (5.5%), Grade ≥ 3 TRAEs (33.3%); hypercholesterolemia (16.7%), increased lipase (11.1%), anemia (11.1%).	[41]
**HRS-4642/** Hengrui Pharma	Dual state (ON/OFF)	IV	Phase 1	NSCLC (*n* = 38), PDAC (*n* = 24)	T1/2: 72 h	78%	800 or 1200 mg daily	Not reached	Grade ≥ 3 TRAEs (23.8%); hypertriglyceridemia (3.6%), decreased neutrophil count (3.6%), hypercholesterolemia (3.6%), death (1.2%).	[42]
**RNK08954/** Ranok Therapeutics (Hangzhou) Co., Ltd. (Hangzhou, Zhejiang, China)	Dual state (ON/OFF)	OA	Phase 1/2	[NSCLC (*n* = 14); PDAC (*n* = 17), ovarian/endometrial cancers (*n* = 3) and others (*n* = 8)	NA	NA	NA	NA	Grade ≥ 3 TRAEs; diarrhea (5%), vomiting (2%), decreased appetite (5%), decreased white blood cells (2%), hypokalemia (5%).	[38]
**Zoldonrasib (RMC-9805)/** Revolution Medicines, Inc.	ON-state (tri-complex inhibitor)	OA	Phase 1/1b	NA	NA	Not observed	1200 mg once daily	Not reached	Grade 3 nausea (1.1%), increased ALT (1.1%), Grade ≥ 2 (≥10%); nausea (39%), vomiting (18%), diarrhea (24%), rash (12%).	[47]
**Zoldonrasib (RMC-9805)/** Revolution Medicines, Inc.	ON-state (tri-complex inhibitor)	OA	Phase 1/1b	PDAC; (*n* = 104, 58%)	NA	Not observed	1200 mg daily	Not reached	Grade 3 increased ALT (1.1%), Grade ≥ 2 (≥10% of patients); nausea (27%), vomiting (15%), diarrhea (20%), rash (10%).	[48]
**INCB161734**/ Incyte (Wilmington, DE, USA)	Dual state (ON/OFF)	OA	Phase 1	PDAC (*n* = 20), CRC (*n* = 12), ovarian cancer (*n* = 2), other (*n* = 2)	NA	Not observed	NA	Not reached	Grade ≥ 2 TRAEs (≥15%); nausea (67%), vomiting (50%), diarrhea (58%), fatigue (22%), increased lipase (17%).	[50]
**GFH375/** GenFleet (Shanghai, China)/Verastem (Needham, MA, USA)	Dual state (ON/OFF)	OA	Phase 1/2	PDAC (*n* = 11), NSCLC (*n* = 11), CRC (*n* = 5), others (*n* = 5)	Tmax: 2–4 h T1/2: 18.5–21.6 h	Not observed	NA	NA	Serious AEs (15.6% of patients); Grade ≥ 3 TRAEs (25%), Grade ≥ 2 TRAEs (≥15%); nausea (62.5%), vomiting (71.9%), diarrhea (71.9%).	[54]
**GFH375/** GenFleet/Verastem	Dual state (ON/OFF)	OA	Phase 1/2	PDAC (*n* = 66)	NA	NA	NA	NA	All grade/≥grade 3 TRAEs; nausea (47%/0), vomiting (47%/1.5%), diarrhea (56%/3%), decreased appetite (33%/3%), asthenia (26%/0), white blood cell count decreased (36%/2%), neutrophil count decreased (49%/8%), anemia (42%/8%), platelet count decreased (29%/2%), hypoalbuminemia (33%/0).	[55]
**Pan-RAS/Pan-KRAS inhibitors**										
**Daraxonrasib (RMC-6236)/** Revolution Medicines	ON-state (tri-complex inhibitor)	OA	Phase 1/2	Advanced solid tumors (including NSCLC and PDAC)	NA	NA	NA	NA	TRAEs (≥10% of patients); rash (52%), diarrhea (21%), nausea (21%), and vomiting (15%). The only grade ≥ 3 TRAE was in a patient with PDAC who had a grade 4 large intestine perforation at the site of an invasive tumor that reduced in size on treatment.	[63]
**BI-2493**	OFF-state	OA	NA	NA	NA	NA	NA	NA	NA	
**BI-2865**/ Boehringer Ingelheim (Ingelheim am Rhein, Germany)	OFF-state	OA	NA	NA	NA	NA	NA	NA	NA	
**BI-1701963/** Boehringer Ingelheim	Indirect inhibitor	OA	Phase 1	Solid tumors (*n* = 28)	Tmax: 0.5–3 h. Cmax: 22.1 nmol/L/mg. AUC0–24 h: 222.6 nmol*h/L/m. Apparent T1/2: 12–26 h	NA	800 mg	NA	TRAEs (64% of patients); most commonly diarrhea, fatigue and decreased platelet count (14%); Grade ≥ 3 TRAEs: hypertension, duodenal obstruction, G4 decreased platelet count. One DLT was reported: G4 decreased platelet count (800 mg).	[65]
**AMG 410/** Amgen (Thousand Oaks, California, USA)	Dual state (ON/OFF)	OA	Phase 1/1b	Advanced solid tumors (including CRC, PDAC, NSCLC)	NA	NA	NA	NA	NA	[66]
**ERAS-0015/** Erasca (San Diego, CA, USA)	ON-state (tri-complex inhibitor)	OA	Phase 1/1b	Advanced solid tumors	NA	NA	NA	NA	NA	[67]

Abbreviations: AE, adverse event; ALT, alanine aminotransferase; AST, aspartate aminotransferase; AUC, area under the curve; BID, twice daily; Cmax, maximum plasma concentration; CRC, colorectal cancer; DLTs, dose-limiting toxicities; GGT, gamma-glutamyl transferase; GI, gastrointestinal; IV, intravenous; MTD, maximum tolerated dose; NA, not available; NSCLC, non-small-cell lung cancer; OA, oral administration; PDAC, pancreatic ductal adenocarcinoma; PK, pharmacokinetics; QD, once daily; T1/2, half-life; TEAE, treatment-emergent adverse event; Tmax, time to peak concentration; TRAE treatment-related adverse event.

**Table 3:** Clinical activity of KRAS G12C inhibitors as monotherapy.

Drug	Trial (NCT)	Phase	Tumor Type	Setting	ORR (%)	mPFS (Months)	mOS (Months)
Sotorasib	CodeBreaK 100 (NCT03600883)	II	NSCLC	Pretreated	37.1	6.8	12.5
Sotorasib	CodeBreaK 100 (NCT03600883)	II	CRC	Pretreated	9.7	4.0	10.6
Sotorasib	CodeBreaK 100 (NCT03600883)	I/II	PDAC	Pretreated	21	4.0	6.9
Adagrasib	KRYSTAL-1 (NCT03785249)	II	NSCLC	Pretreated	42.9	6.5	12.6
Adagrasib	KRYSTAL-1 (NCT03785249)	II	CRC	Pretreated	23.3	5.6	19.8
Adagrasib	KRYSTAL-1 (NCT03785249)	II	PDAC	Pretreated	33.3	5.4	8.0
Divarasib	NCT04449874	I	NSCLC	Pretreated	55.6	13.8	–
Divarasib	NCT04449874	I	CRC	Pretreated	29.1	5.6	–
Glecirasib	NCT05009329	II	NSCLC	Pretreated	47.9	8.2	–
Fulzerasib	NCT05005234	II	NSCLC	Pretreated	49.1	9.7	–
Garsorasib	NCT05383898	II	NSCLC	Pretreated	50.0	7.6	–

Abbreviations: CRC, colorectal cancer; mOS, median overall survival; mPFS, median progression-free survival; NCT, clinical trial number; NSCLC, non-small-cell lung cancer; ORR, objective response rate; PDAC, pancreatic ductal adenocarcinoma.

**Table 4:** Clinical activity of KRAS G12C inhibitors in combination therapies.

Drug + Combination	Trial (NCT)	Phase	Tumor Type	Setting	ORR (%)	mPFS (Months)
Sotorasib + Panitumumab	CodeBreaK 300 (NCT05198934)	III	CRC	Pretreated	30.2	5.6
Sotorasib + Panitumumab	CodeBreaK 101 (NCT04185883)	I	CRC	Pretreated	30	5.7
Adagrasib + Cetuximab	KRYSTAL-1 (NCT04793958)	I/II	CRC	Pretreated	34.0	6.9
Divarasib + Cetuximab	NCT04449874	I	CRC	Pretreated	62.5	~8.1
Garsorasib + Cetuximab	NCT04585035	I/II	CRC	Pretreated	45.2	7.5
Glecirasib + Cetuximab	NCT05194995	I/II	CRC	Pretreated	50	6.8
Sotorasib + Panitumumab + FOLFIRI	CodeBreaK 101 (NCT04185883)	I	CRC	Pretreated	57.5	8.2
Sotorasib + Pembrolizumab/Atezolizumab	CodeBreaK 101 (NCT04185883)	I	NSCLC	Pretreated	29	–
Adagrasib + Pembrolizumab	KRYSTAL-7 (NCT04613596)	II	NSCLC	First-line	61	27.7

Abbreviations: CRC, colorectal cancer; FOLFIRI, chemotherapy consisting of folinic acid, fluorouracil, and irinotecan; mPFS, median progression-free survival; NCT, clinical trial number; NSCLC, non-small-cell lung cancer; ORR, objective response rate.

## Data Availability

No new data were created or analyzed in this study. Data sharing is not applicable to this article.

## Abbreviations

The following abbreviations are used in this manuscript:

AE	Adverse Event
ALT	Alanine Aminotransferase
AST	Aspartate Aminotransferase
AUC	Area Under the Curve
BID	Twice Daily
Cmax	Maximum Plasma Concentration
CRC	Colorectal Cancer
CYP	Cytochrome P450
DLTs	Dose-Limiting Toxicities
ECOG PS	Eastern Cooperative Oncology Group Performance Status
EMT	Epithelial-to-Mesenchymal Transition
GAP	GTPase-Activating Protein
GDP	Guanosine Diphosphate
GEF	Guanine Nucleotide-Exchange Factor
GGT	Gamma-Glutamyl Transferase
GI	Gastrointestinal
GTP	Guanosine Triphosphate
HRAS	Harvey Rat Sarcoma
ILD	Interstitial Lung Disease
IV	Intravenous
KRAS	Kirsten Rat Sarcoma
mOS	Median Overall Survival
mPFS	Median Progression-Free Survival
MTD	Maximum Tolerated Dose
NA	Not Available
NCT	Clinical Trial Number
NRAS	Neuroblastoma Rat Sarcoma
NSCLC	Non-Small-Cell Lung Cancer
OA	Oral Administration
ORR	Objective Response Rate
OS	Overall Survival
PDAC	Pancreatic Ductal Adenocarcinoma
PD-L1	Programmed Death-Ligand 1
PFS	Progression-Free Survival
P-gp	P-Glycoprotein
PK	Pharmacokinetics
PPIs	Proton Pump Inhibitors
QD	Once Daily
QTc	Corrected QT Interval
RAS	Rat Sarcoma
T1/2	Half-Life
TEAE	Treatment-Emergent Adverse Event
TESAE	Treatment-Emergent Serious Adverse Event
Tmax	Time to Peak Concentration
TME	Tumor Microenvironment
TRAE	Treatment-Related Adverse Event
TRSAE	Treatment-Related Serious Adverse Event
Vd	Volume of Distribution
